# *STP10* encodes a high-affinity monosaccharide transporter and is induced under low-glucose conditions in pollen tubes of Arabidopsis

**DOI:** 10.1093/jxb/erw048

**Published:** 2016-02-18

**Authors:** Theresa Rottmann, Wolfgang Zierer, Christa Subert, Norbert Sauer, Ruth Stadler

**Affiliations:** Molecular Plant Physiology, University Erlangen-Nürnberg, 91058 Erlangen, Germany

**Keywords:** Arabidopsis, gene expression regulation, glucose, hexokinase, monosaccharides, pollen tubes, radioactive uptake measurement, *Saccharomyces cerevisiae*, STP10, sugar signalling, sugar transport.

## Abstract

Pollen tubes are fast growing, photosynthetically inactive cells. Their energy demand is covered by specific transport proteins in the plasma membrane that mediate the uptake of sugars. Here we report on the functional characterization of AtSTP10, a previously uncharacterized member of the SUGAR TRANSPORT PROTEIN family. Heterologous expression of *STP10* cDNA in yeast revealed that the encoded protein catalyses the high-affinity uptake of glucose, galactose and mannose. The transporter is sensitive to uncouplers of transmembrane proton gradients, indicating that the protein acts as a hexose–H^+^ symporter. Analyses of *STP10* mRNA and *STP10* promoter–reporter gene studies revealed a sink-specific expression pattern of *STP10* in primordia of lateral roots and in pollen tubes. This restriction to sink organs is mediated by intragenic regions of *STP10*. qPCR analyses with cDNA of *in vitro* grown pollen tubes showed that *STP10* expression was down-regulated in the presence of 50mM glucose. However, in pollen tubes of glucose-insensitive plants, which lack the glucose sensor hexokinase1 (HXK1), no glucose-induced down-regulation of *STP10* expression was detected. A *stp10* T-DNA insertion line developed normally, which may point towards functional redundancy. The data presented in this paper indicate that a high-affinity glucose uptake system is induced in growing pollen tubes under low glucose conditions and that this regulation may occur through the hexokinase pathway.

## Highlight

STP10 is part of a high-affinity monosaccharide uptake system in the plasma membrane of pollen tubes of Arabidopsis. It is down-regulated under high-glucose conditions, possibly through the hexokinase pathway.

## Introduction

In Arabidopsis and many other herbaceous plants, photosynthetically fixed carbon is used to synthesize sucrose, which is distributed within the plant body via the phloem. Young leaves, roots, meristems and reproductive tissues are so-called sink organs that depend on a supply of nutrients. In such sink tissues, plasmodesmata connect sieve elements of the phloem and adjacent cells. Hence, sucrose can move from the phloem through plasmodesmata into the cells of these sink tissues without leaving the cytosol (Patrick, 1997; Imlau *et al.*, 1999; Turgeon and Wolf, 2009). However, there are some tissues or cell types that are symplastically isolated, for example the inner integument of the seed coat, the endosperm and the embryo (Stadler *et al.*, 2005) as well as egg cells (Werner *et al.*, 2011) and pollen grains (Scott *et al.*, 1991). Here, the energy supply involves an apoplasmic step in which sucrose is first exported into the apoplast and subsequently imported into the sink cells by specific transport proteins. The export step is mediated by SWEET proteins (*S*UGARS *W*ILL *E*VENTUALLY BE *E*XPORTED *T*RANSPORTERS), which are localized in the plasma membrane and transport sucrose or glucose along an existing concentration gradient (Chen *et al.*, 2010; Chen *et al.*, 2012). Sucrose transporting SWEETs have been localized in the seed coat and in the endosperm (Chen *et al.*, 2015). SWEET8/RUPTURED POLLEN GRAIN1 (RPG1) exports glucose out of the tapetum cells that surround the developing pollen grains in the anthers (Guan *et al.*, 2008; Sun *et al.*, 2013). Sugar uptake from the apoplast into sink cells is catalysed by sucrose transporters, or, if extracellular invertases are present, by monosaccharide transporters (Sauer, 2007). In Arabidopsis a family of nine *SUC*ROSE TRANSPORT proteins (AtSUC1–9; Sauer *et al.*, 2004) exists and a family of 14 homologous monosaccharide transporters (*S*UGAR *T*RANSPORT *P*ROTEIN, AtSTP1-14; Büttner *et al.*, 2000) has been identified. STPs are members of the Arabidopsis *M*ONO*S*ACCHARIDE *T*RANSPORTER MST(-like) superfamily. The MST(-like) family includes 53 monosaccharide transporters, subdivided into seven individual families (Büttner, 2007). Amongst them, the *AtSTPs* constitute the best-characterized group. Nine *STP* genes have been characterized in detail so far. Their expression is restricted to sink tissues or symplastically isolated cells like guard cells with the exception of *STP3* and *STP14* (Büttner *et al.*, 2000; Poschet *et al.*, 2010). These large sugar transporter families allow a fine-tuned regulation of sugar supply adjusted to the type of sink tissue, developmental stage, metabolic state and environmental conditions. Especially for pollen tubes an adapted energy supply from the surrounding maternal apoplast seems to be essential as their tip growth is one of the fastest plant growth processes and efficient tube elongation is crucial to the reproductive success of the plant. The need for sugar uptake into the male gametophyte is underlined by the identification of sucrose transport proteins and invertases in pollen tubes of several plant species (Stadler *et al.*, 1999; Ylstra *et al.*, 1998; Lemoine *et al.*, 1999; Maddison *et al.*, 1999; Meyer *et al.*, 2004). Different analyses revealed that additionally at least five *STP*s are expressed specifically or preferentially in pollen: *STP2* mRNA and protein could be detected only during the first stages of pollen development (Truernit *et al.*, 1999) whereas *STP4*, *STP6*, *STP9*, and *STP11* are expressed mainly in the later stages of the microgametogenesis and/or in growing pollen tubes (Truernit *et al.*, 1996; Scholz-Starke *et al.*, 2003; Schneidereit *et al.*, 2003; Schneidereit *et al.*, 2005; Büttner, 2010). Microarray data suggest that *STP11* is the most strongly expressed *STP* in growing pollen tubes (Wang *et al.*, 2008; Qin *et al.*, 2009). The same microarray data furthermore indicate a rather high expression of the so far uncharacterized *STP10* (*At3g19940*) in pollen tubes.

In the present paper, we describe the detailed characterization of the Arabidopsis monosaccharide transporter gene *STP10*, which is expressed in pollen tubes and also in emerging side roots. The transport properties of the encoded STP10 protein were analysed in baker’s yeast. A T-DNA insertion line for *STP10* did not show any phenotypical difference compared with the wild type (WT). The potential physiological role of STP10 for pollen tubes is discussed.

## Materials and methods

### 

#### Strains, growth conditions and genotyping

*Arabidopsis thaliana* (L.) Heynh. (ecotype Col-0) was grown under long day conditions (16h light–8h dark) at 22 °C and 60% relative humidity or in the greenhouse in potting soil. Plants used for the generation of protoplasts were grown under a short day regime (8h light–16h dark). For the analysis of seedlings or roots, seeds were cultivated on MS plates (Murashige and Skoog, 1962). The T-DNA insertion lines *stp10-1* (SALK_207063; Alonso *et al.*, 2003) and SALK_070739 (Alonso *et al.*, 2003; Aki *et al.*, 2007; Hsu *et al.*, 2014) were obtained from the Nottingham Stock Centre (http://arabidopsis.info/). The primers STP10g+1762r (5′-ATTGGTATTGTTGTCATCATGTCCACC-3′) and SALK_LBb1.3 (5′-ATTTTGCCGATTTCGGAAC-3′) were used in a PCR to detect the mutant allele, STP10g+661f (5′-GGAACATCAAAGATGGCTCAACATG-3′) and STP10g+1762r to detect the WT allele. Segregation analysis of the *stp10-1* allele was performed by PCR-based genotyping with the same primer pairs. The position of the T-DNA insertion was determined by sequencing a PCR product obtained from *stp10-1* genomic DNA with the primer pair STP10g+1762r/SALK_LBb1.3. The *glucose insensitive2-1* (*gin2-1*) mutant line (Moore *et al.*, 2003) was kindly provided by Jen Sheen (Department of Molecular Biology, Massachusetts General Hospital). Arabidopsis was transformed via floral dip with *Agrobacterium tumefaciens* Smith & Townsend strain GV3101 (Holsters *et al.*, 1980; Clough and Bent, 1998). *Escherichia coli* (Migula) Castellani & Chalmers strain DH5α (Hanahan, 1983) was used for all cloning steps. Heterologous expression analysis was performed in *Saccharomyces cerevisiae* Meyen *ex* E.C. Hansen strain CSY4 000 (see below).

#### RNA isolation, RT–PCR and qPCR

Total RNA was isolated using the InviTrap® Spin Plant RNA Mini Kit 0711 (STRATEC). For RNA isolation from pollen tubes, pollen of about 30 flowers was germinated *in vitro* or semi-*in vivo* (see below) for at least 5h. Pollen tubes were collected by vortex-mixing the cellulosic membrane in 100 µl extraction buffer of the PicoPure^TM^ RNA Isolation Kit (Arcturus). RNA isolation was carried out according to the PicoPure^TM^ manual. The QuantiTect® Reverse Transkription Kit (Qiagen) was used for the reverse transcription reaction of about 300ng RNA each. PCR was carried out with primer pair STP10g+661f (5′-GGAACATCAAAGATGGCTCAACATG-3′) and STP10g+1762r (5′-ATTGGTATTGTTGTCATCATGTCC ACC-3′) to detect the *STP10* transcript or with primers AtACT2g+846f (5′-ATTCAGATGCCCAGAAGTCTTGTT-3′) and AtACT2g+1295r (5′-GAAACATTTTCTGTGAACGATTCCT-3′) to detect the *Actin2* transcript. Relative expression of *STP10* and *STP4* in pollen tubes was measured via qRT-PCR using the SYBR® Green Kit (Agilent, Santa Clara, CA, USA) and the Rotor-Gene Q Cycler (Qiagen). Primers STP10c+1057f (5′-ATGTTCATTTGTCAGCTTCTTGTTGGT TCTT-3′) and STP10c+1305r (5′-TTGTCAAAAAGAATTGACC AATGAGAAAAGTGAAG-3′) were used for the detection of *STP10* transcripts, STP4c+1071f (5′-GCTTGTCTCTCAGA TCGCTATTGGA-3′) and STP4c+1247r (5′-GAGCTG CTGATCGGATCTCTAGTG-3′) for *STP4*. Relative *STP* expression levels were normalized by comparison with *UBI10* which was amplified with primer pair UBQ10+1066f (5′-GATGGTCGTACTT TGGCGGATTAC-3′) and UBQ10+1130r (5′-AGACGCAACA CCAAGTGAAGGG-3′) and calculated according to the 2^–ΔΔCT^ (Livak) method.

#### Cloning of Gateway® destination vectors

For convenient selection of transgenic promotor–gene-reporter lines, Gateway®-compatible destination vectors carrying the glufosinate resistance gene (*bar*) were generated. To create pBASTA-GFP the Gateway® cassette and *GFP* gene from pMDC107 (Curtis and Grossniklaus, 2003) were amplified with the primers Gateway+GFPf+SbfI (5′-CCTGCAGGATTCCCG ATCTAGTAACATAGATGACACCG-3′) and Gateway+GFPr+PacI (5′-TTAATTAAGTACCGAGCTCGAATTATCACAAGTTTG-3′). This resulting 2796bp fragment and all other PCR fragments of the present work were confirmed by sequencing. The fragment was cloned via *Sbf*I/*Pac*I into the binary plant expression vector AKK1472 (Collier *et al.*, 2005). For pBASTA-GUS, the Gateway® cassette and *GUS* gene from pMDC163 (Curtis and Grossniklaus, 2003) were amplified with the primers Gateway+GUSf+XhoI (5′-CTCGAGTGTGG AATTGTGAGCGGATA-3′) and Gateway+GUSr+XhoI (5′-CTCGAG GTTTTCCCAGTCACGACGTT-3′). The resulting 4041bp fragment was cleaved with *Xho*I and ligated into AKK1472. In order to obtain a glufosinate resistance gene containing Gateway® destination vector that allows the integration of a construct in front of a Nos terminator, the vector pMDC123 (Curtis and Grossniklaus, 2003) was modified as follows: The Nos terminator of pMDC43 (Curtis and Grossniklaus, 2003) was amplified with the primers NosT_f+SpeI+CACC (5′-CACCACTAGTAGTAACATAGATGACAC-3′) and NosT_r+SpeI (5′-ACTAGTGAATTTCCCCGATCG-3′). The resulting 274bp fragment was ligated into the plant expression vector pMDC123 via *Spe*I yielding the new vector pMDC123_nosT.

#### Cloning of reporter gene constructs for STP10

For the *pSTP10:STP10g-reporter* plants a 2830bp fragment was amplified with the primer pair STP10g-1064f+CACC (5′-CACCCGCTTT ATGCAAGAAACAAGAATAGTCA-3′) and STP10c+1542r (5′-ATTGGTATT GTTGTCATCA TGTCCACC-3′), cloned into pENTR/D-TOPO (Invitrogen) and inserted upstream of the *GUS*- or *GFP*::nos terminator box by the LR reaction in pBASTA-GUS or pBASTA-GFP yielding pTR92 and pTR93, respectively. For reporter plants expressing *GUS* without the genomic sequence of *STP10* under the control of the *STP10* promoter, the 1064bp promoter sequence was amplified with primers STP10g-1064f+HindIII (5′-TAAAGCTTCGCTTTATGCAAGAAACAAGA ATAGTCA-3′) and STP10g-1r+AscI (5′-ATGGCGCGCCC TTTTTTTTTCTTGCCT TTGGTCTTAGA-3′) and inserted into the Gateway® vector pMDC123_nosT in front of the attachment site AttR1 via the added *Hin*dIII/*Asc*I sites. The coding sequence for *GUS* was then inserted via the LR reaction from pENTR-GUS yielding plasmid pTR112.

For the subcellular localization of STP10, fusion constructs with *GFP* under the control of the 35S promoter were generated. For the *STP10c-GFP* fusion (pTR148), the coding sequence (CDS) of *STP10* was amplified from cDNA obtained from semi-*in vivo* grown pollen tubes as template with the primer pair STP10c+1f+CACC (5′-CACCATGGCAGGA GGAGCTTTTGTATCAG-3′) and STP10c+1542r (5′-ATTGGTA TTGTTGTCATCAT GTCCACC-3′). For the *GFP-STP10c* fusion (pTR147) the reverse primer STP10c+1545rev (5′-TTAATTGGT ATTGTTGTCATCATGTCCAC-3′) was used to include the STOP codon. Both PCR fragments were cloned into TOPO/pENTR (Invitrogen) and then inserted into pMDC43 (Curtis and Grossniklaus, 2003) for *GFP-STP10c* or pMDC83 (Curtis and Grossniklaus, 2003) for *STP10c-GFP*.

#### Isolation and transformation of protoplasts

Leaf mesophyll protoplasts were generated from Col-0 plants as described by Drechsel *et al.* (2011) and transformed via the polyethylene glycol method (Abel and Theologis, 1994). Transformed protoplasts were incubated for 40–72h in the dark at 22 °C prior to confocal analysis.

#### Microscopy

GUS plants were analysed under a stereomicroscope (Leica MZFLIII; Leica Microsystems) or a microscope (Zeiss Axioskop; Carl Zeiss Jena GmbH). Images were processed using the analySIS Doku 3.2 software (Soft Imaging System, Münster, Germany).

Images of protoplasts and GFP-reporter plants were taken on a confocal laser scanning microscope (Leica TCS SPII; Leica Microsystems) using a 488nm argon laser for excitation and processed with Leica Confocal Software 2.5. Detection windows ranged from 497 to 526nm for GFP and from 682 to 730nm for chlorophyll autofluorescence.

#### Functional characterization of STP10 by heterologous expression

In order to generate a heterologous expression system that can be used for the analysis of both sucrose and monosaccharides transporters, the hexose-transport- and invertase-deficient *S. cerevisiae* strain CSY4 000 was constructed. To this end the deletion cassette of pUG73 (Gueldener *et al.*, 2002) was amplified with the primer pair SUC2delta5 (5′-AAATAGATATGTATTATTCTTCAAAACATTCTCTT GTTCTTGTGCGCATAGGCCACTAGTGGATCTG-3′) and SUC2delta3 (5′-GTTTTACATTCGTCACTCGTTAGCTAAAGC CCTTTAGAATGGCTTCAGCTGAAGCTTCGT ACGC-3′). The primers were designed to attach the 3′ and 5′ flanking sequences of the yeast invertase gene *ScSUC2* to the amplified deletion cassette. After direct transformation (Soni *et al.*, 1993) of the PCR product into the hexose-transport-deficient strain EBY.VW4 000 (Wieczorke *et al.*, 1999), these sequences led to the substitution of the *ScSUC2* gene by the *LEU2* gene of the deletion cassette via homologous recombination. Positive clones were identified by PCR with primer pair ScSUC2g-165f (5′-GATCCTATAATCCTTCCTCCTGAAAAG AAACA-3′) and LEU2MX5′out (5′-CAGAACCGGTGACC TTGGTGG-3′). Primers ScSUC2g-165f and ScSUC2g-5′out (5′-TTGGGTTGTATTGAAAGTACAGATGCCATTTG-3′) were used as a control for remaining WT alleles.

The CDS of *STP10* was amplified from pTR147 with the primers STP10c+1f+NotI+YES (5′-TAGCGGCCGCAAGCTTGTAAAA GAAATGGCAGGAGGAGCTTTTGTATCAG-3′) and STP10c+ 1545r+NotI (5′-TAGCGGCCGCTTAATTGGTATTGTTGTCAT CATGTCCAC-3′) that introduced a *Not*I site on both sites of the PCR product as well as the sequence 5′-AAGCTTGTAAAAGAA-3′ (part of the *STP1* 5′UTR) (Stadler *et al.*, 1995) upstream of the start codon. The *STP10* CDS fragment was ligated into the *Not*I site of the vector NEV-N (Sauer and Stolz, 1994), in both sense and antisense orientation, yielding constructs pTR91 and pTR94, respectively. Both constructs were then used to transform CSY4 000, yielding strains TRY1004 (sense) and TRY1005 (antisense). For uptake experiments with ^14^C-labelled sugars, yeast strains were precultured in maltose–casamino acids medium [0.67% (w/v) yeast nitrogen base, 1% (w/v) casamino acids, 0.01% (w/v) Trp and 2% (w/v) maltose] to an *A*_600_ of 1, and transport tests were performed as described by Sauer and Stadler (1993).

#### Pollen germination assays

*In vitro* pollen germination for RNA extraction and growth analysis was done as described (Rodriguez-Enriquez *et al.*, 2012), but only 200mM sucrose was added to the medium. The same medium was used for semi*-in vivo* pollen germination, which was performed by pollinating stigmata, cutting them off and placing them horizontally onto the cellulosic membrane of the growth medium to allow the outgrowth of the pollen tubes from the cut surface (Palanivelu and Preuss, 2006). Pollen tube length was measured with a self-written half automatic program in Python (Python Software Foundation, Beaverton, OR, USA) and plotted with Matplotlib (Hunter, 2007), which was also used for all other graphs. Pollen germination rate was counted using ImageJ 1.47 (Schneider *et al.*, 2012).

## Results

### Sequence analysis of *STP10*

The *STP10* CDS was amplified from total mRNA of pollen tubes. Comparison with the genomic sequence confirmed the predicted exon/intron structure with three exons separated by two introns (Fig. 7A). Five possible intron positions are known in the *STP* gene family, of which positions 1, 2 and 5 are highly conserved (Büttner *et al.*, 2000). The introns of *STP10* correspond to positions 2 and 5, and hence *STP10* is the only member of the family not having an intron at position 1. The protein encoded by *STP10* comprises 514 amino acids and has a calculated molecular mass of 56.2kDa and an estimated isoelectric point of 8.0 (http://isoelectric.ovh.org/). The STP10 protein is predicted to have 12 transmembrane domains like all other members of the STP family. The sequence includes three Asn–X–Ser consensus sequences for N-glycosylation, but two of them are localized within transmembrane domains and are, therefore, most likely not glycosylated. Although *STP10* and *STP4* evolved by tandem gene duplication (Johnson and Thomas, 2007) and are direct neighbours on chromosome 3, their encoded proteins only display 64% identical and 79% similar amino acids. The closest relative of *STP10* is *STP9* on chromosome 1, with 85% identical and 95% similar positions, followed by *STP11* and *STP4*.

### Analysis of *STP10* expression in flowers

**Fig. 1.**
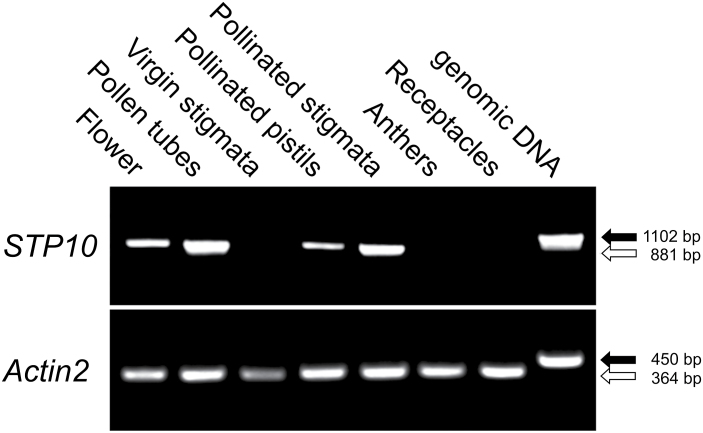
RT-PCR analysis of *STP10* expression in different floral tissues. Total RNA preparations from whole flowers, *in vitro* germinated pollen tubes, virgin stigmata, pollinated pistils, semi-*in vivo* grown pollen tubes (pollinated stigmata), anthers and receptacles with nectaries were tested for *STP10* expression with primers specific for *STP10*. Arrows indicate the size of PCR products derived from reverse-transcribed mRNA (white) and genomic DNA (black). The presence of RNA in each sample was confirmed with *Actin2*-specific primers.

To investigate the predicted expression of *STP10* in pollen tubes and to screen for *STP10* expression in other floral tissues, RT-PCRs were performed on RNA preparations isolated from whole open flowers, *in vitro* grown pollen tubes, virgin stigmata, pollinated pistils, semi-*in vivo* grown pollen tubes including the stigmata, anthers and receptacles with the nectaries. A PCR reaction with *Actin2*-specific primers served as a control for the presence of intact cDNA in each sample. As shown in Fig. 1, a PCR product could be amplified from whole flowers with *STP10*-specific primers. After 36 PCR cycles no product could be obtained from virgin stigmata, anthers or the receptacle indicating that there is no expression of *STP10* in these parts of the flower. From pollinated pistils and pollen tubes grown *in vitro* or semi-*in vivo*, the *STP10*-specific fragment could be amplified. As virgin stigmata showed no *STP10* expression, the PCR product in pollinated pistils and semi-*in vivo* grown pollen tubes probably did not originate from maternal tissues, but from the pollen tubes in these RNA preparations.

### Reporter gene analysis of *STP10* expression

**Fig. 2.**
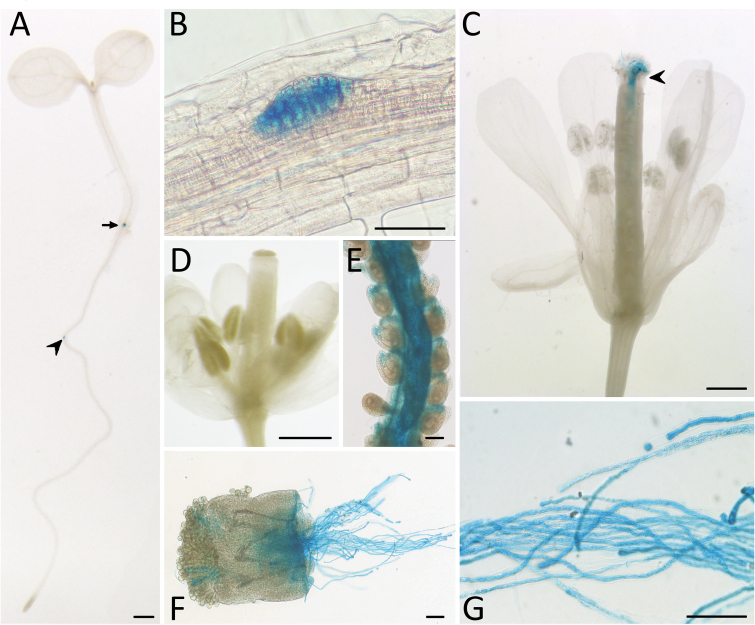
*STP10* promoter activity in *pSTP10*:*STP10g*-*GUS* reporter plants. Histochemical detection of β-glucuronidase activity in Arabidopsis Col-0 expressing a *pSTP10:STP10g-GUS* fusion construct. (A) Seven-day-old seedling with GUS staining in an emerging side root (arrowhead) and the adventitious root meristem (arrow). (B) Side root bud at a higher magnification. (C) Pollinated flower. The arrowhead indicates pollen tubes growing through the ovary. (D) Non-pollinated flower. (E) Peeled ovaries with GUS-positive pollen tubes in the transmitting tract and the funiculi. (F, G) Pollen tubes grown semi-*in vivo* (F) or *in vitro* (G). Scale bars: 1mm in (A); 500 µm in (C, D); 50 µm in (B, E, F, G).

**Fig. 3.**
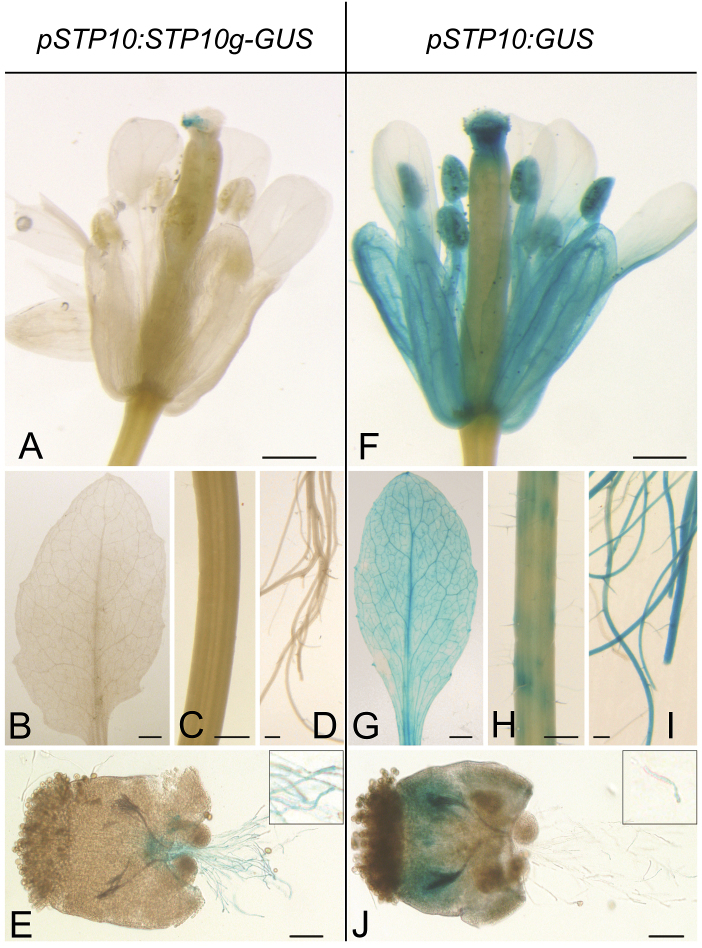
Comparison of *GUS* expression in *pSTP10:STP10g-GUS* and *pSTP10:GUS* reporter plants. Detection of β-glucuronidase activity in Arabidopsis Col-0 expressing a *pSTP10:STP10g-GUS* fusion construct (A–E) or merely *pSTP10:GUS* (F–I) in flowers (A, F), rosette leaves (B, G), stems (C, H), roots (D, I) and semi-*in vivo* grown pollen tubes (E, J). Inset in (E, J): higher magnification of pollen tubes. All tissues were stained for the same time for both reporter plant types. Scale bars: 500 µm in (A, C, D, F, H, I); 2.5mm in (B, G); 100 µm in (E, J).

To examine the *STP10* expression in detail, transgenic Arabidopsis plants were generated. *pSTP10:STP10g-GFP* and *pSTP10:STP10g-GUS* plants driving reporter gene expression from a 1064bp promoter fragment were obtained by agrobacteria-mediated transformation of Col-0 WT plants with the vector pTR92 (*GUS*) or pTR93 (*GFP*). Of the transformants obtained, nine *pSTP10:STP10g-GFP* lines and six *pSTP10:STP10g-GUS* lines were analysed. In non-floral tissues GUS staining could be observed only in roots (Fig. 2A), where it was restricted to the adventitious root meristem and emerging lateral roots (Fig. 2B). Older roots showed no GUS activity (Fig. 3D).

**Fig. 4.**
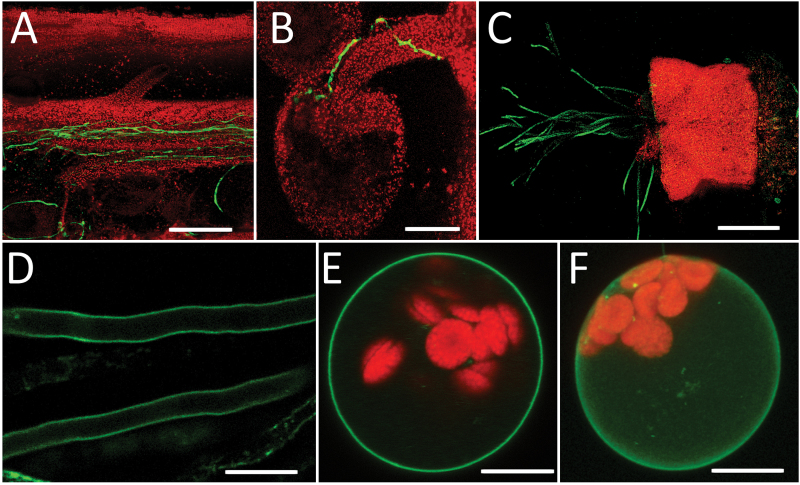
*STP10* promoter activity and subcellular localization of STP10. (A–D) Detection of GFP fluorescence (green) in *pSTP10:STP10-GFP* reporter plants. (A) Partially opened style with pollen tubes growing in the transmitting tract. (B) Pollen tubes growing along the funiculus towards an ovule. (C) Semi-*in vivo* germinated pollen tubes. (D) Optical section through pollen tubes. (E, F) Single optical section (E) and maximum projection (F) of a mesophyll protoplast expressing *GFP-STP10c* under the control of the 35S promoter. Chlorophyll autofluorescence is shown in red. Scale bars: 100 µm in (A); 150 µm in (B); 50 µm in (C); 10 µm in (D, E, F).

All other vegetative tissues showed no GUS staining. In flowers a blue staining could be detected in pollinated pistils (Fig. 2C) and the staining was even more intense when the pistil was opened prior to staining (Fig. 2E). Strong GUS activity was detectable in pollen tubes grown *in vitro* (Fig. 2G) or semi*-in vivo* (Fig. 2F). The intense GUS activity in pollen tubes led to the hypothesis that the GUS staining in pistils (Fig. 2C/E) originated from the pollen tubes growing through and not from the maternal tissue itself. This hypothesis was confirmed by the absence of GUS staining in unpollinated flowers (Fig. 2D) and the analysis of plants expressing *GFP* as a reporter gene (Fig. 4). GFP fluorescence in the pistil originated only from pollen tubes growing through the transmitting tract (Fig. 4A) and along the funiculi (Fig 4B).

This finding is consistent with the absence of *STP10* mRNA in virgin stigmata and its presence in pollinated stigmata shown by RT-PCR. The absence of *STP10* expression in anthers confirmed both by RT-PCR and reporter gene analysis indicated that *STP10* expression is induced during pollen germination and that the STP10 protein is localized exclusively in growing pollen tubes.

Interestingly, in reporter plants expressing *GUS* directly under the control of the *STP10* promoter (*pSTP10:GUS*), GUS staining was not restricted to pollen tubes and side root meristems (Fig. 3). Plants expressing *GUS* without the genomic sequence of *STP10* showed a strong GUS activity also in whole roots, leaves, stems, sepals, anthers, and stigmata (Fig. 3F–I). However, the GUS staining of pollen tubes (Fig. 3J) in all 12 analysed plants was weaker compared with all tested *pSTP10:STP10g-GUS* plants. The presence of the genomic *STP10* sequence therefore seems to be necessary for both restricting the *GUS* expression to pollen tubes and side root buds and enhancing its expression in pollen tubes.

Optical sections of pollen tubes (Fig. 4C) suggested that STP10-GFP is localized at the plasma membrane of the pollen tubes (Fig. 4D). To further analyse the subcellular localization, *STP10-GFP* and *GFP-STP10* fusion constructs were expressed in Arabidopsis protoplasts under the control of the 35S promoter. In single optical sections (Fig. 4E) and maximum projections (Fig. 4F) the GFP-STP10 fusion protein labelled the plasma membrane. STP10-GFP showed the same subcellular localization (Supplementary Fig. S1 at *JXB* online), characterizing STP10 as a plasma membrane protein.

### Functional characterization of STP10 by heterologous expression in yeast

**Fig. 5.**
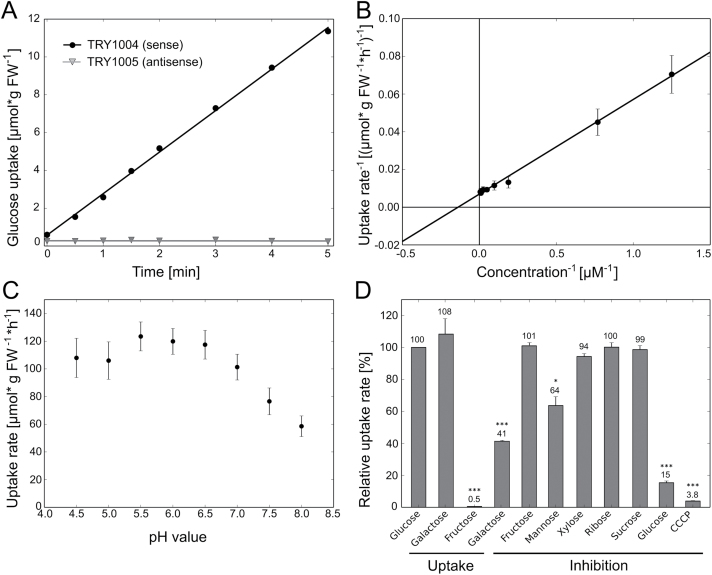
Characterization of STP10 transport activity in transgenic baker′s yeast. (A) Uptake of [^14^C]glucose into yeast strains TRY1004 (expressing *STP10* in sense orientation, circles) and TRY1005 (control strain expressing *STP10* in antisense orientation; triangles) per gram fresh weight (FW) at an initial outside concentration of 100 µM glucose at pH 5.5. (B) Uptake rates for increasing concentrations of [^14^C]glucose were determined for the calculation of the *K*_M_ value for D-glucose uptake of the *STP10*-expressing yeast strain TRY1004 according to Lineweaver–Burk. The plot represents mean values of at least three biological replicates for each sugar concentration including standard deviations. (C) Uptake rate of [^14^C]glucose into TRY1004 at different pH values at an initial outside concentration of 20 µM glucose. (D) Determination of the substrate specificity and sensitivity to uncouplers of STP10. Relative uptake rates of [^14^C]galactose and [^14^C]fructose were measured at an initial outside concentration of 100 µM at pH 5.5. Transport activity of STP10 for other sugars was determined by competitive inhibition of glucose uptake (10 µM initial outside concentration) in the presence of non-radioactive sugars in 10-fold excess. The addition of 100 µM cold glucose was used as a control. The uncoupler CCCP was added to a final concentration of 50 µM. Data represent means and standard errors (SE) of three independent biological replicates.*P≤0.05, ***P≤0.001 by Student’s *t*-test.

To investigate the transport properties of the encoded protein, *STP10* was expressed in a hexose-transport- and invertase-deficient yeast mutant. This novel yeast strain was generated from the hexose-transport-deficient strain EBY.VW4000 (Wieczorke *et al.*, 1999) by deleting the invertase gene (Δ*suc2*) and inserting the *LEU2* gene from *Klyveromyces lactis* that complemented the *leu2* mutation of EBY.VW4 000. The resulting strain CSY4 000 constitutes a useful heterologous expression system for sugar transporters and allows testing of their uptake properties for both sucrose and monosaccharides in one yeast strain. To analyse this for *STP10* its CDS was amplified by PCR and cloned into the yeast expression vector NEV-N (Sauer and Stolz, 1994). The forward primer extended the N-terminal sequence for a part of the *STP1* 5′UTR in front of the start codon. This sequence is reported to optimize the expression of plant genes in baker’s yeast (Stadler *et al.*, 1995). As shown in Fig. 5A, the yeast strain expressing *STP10* in sense orientation (TRY1004) regained the ability to accumulate [^14^C]glucose, while expression of the *STP10* gene in antisense orientation (TRY1005) did not restore the uptake. TRY1004 was used to determine the *K*_M_ value, pH optimum and substrate specificity of STP10. The *K*_M_ of STP10 for glucose was measured to be 7.6±1.7 µM (Fig. 5B), which is the lowest value of all STPs characterized so far (Sauer *et al.*, 1990; Truernit *et al.*, 1996; Truernit *et al.*, 1999; Büttner *et al.*, 2000; Scholz-Starke *et al.*, 2003). The maximum uptake rate (*V*_max_) for glucose was 149±24 µmol h^−1^ gFW^−1^. The pH optimum for glucose uptake was rather broad with a peak at pH 5.5 (Fig. 5C), which is consistent with pH optima reported for other STP family members. It was directly shown by uptake measurements with the respective radioactive labelled sugars that STP10 transports glucose and galactose at similar rates, but almost no uptake of [^14^C]fructose could be detected (Fig. 5D). Possible uptake of other sugars was tested by measuring the uptake of [^14^C]glucose in the presence of an excess of non-radioactive sugars. The pentoses xylose and ribose as well as the disaccharide sucrose did not interfere with glucose uptake (Fig. 5D). Mannose reduced the uptake rate of [^14^C]glucose to 64%, indicating that it is transported, but at a lower rate than glucose and galactose, which led to a reduction of [^14^C]glucose uptake to 15% and 41%, respectively, when added in the non-radioactive form. Non-radioactive fructose did not interfere with glucose uptake. Furthermore, glucose uptake decreased significantly in the presence of carbonyl cyanide *m*-chlorophenyl-hydrazone (CCCP), an uncoupler of transmembrane proton gradients, suggesting that sugar uptake via STP10 is driven by a proton gradient across the plasma membrane, as has been shown for other STPs (Sauer *et al.*, 1990; Truernit *et al.*, 1996; Truernit *et al.*, 1999; Büttner *et al.*, 2000; Scholz-Starke *et al.*, 2003; Schneidereit *et al.*, 2005). Taken together, these results indicate that STP10 is an energy-dependent, high affinity hexose–H^+^ symporter.

### Glucose-mediated regulation of *STP10* expression in pollen tubes

**Fig. 6.**
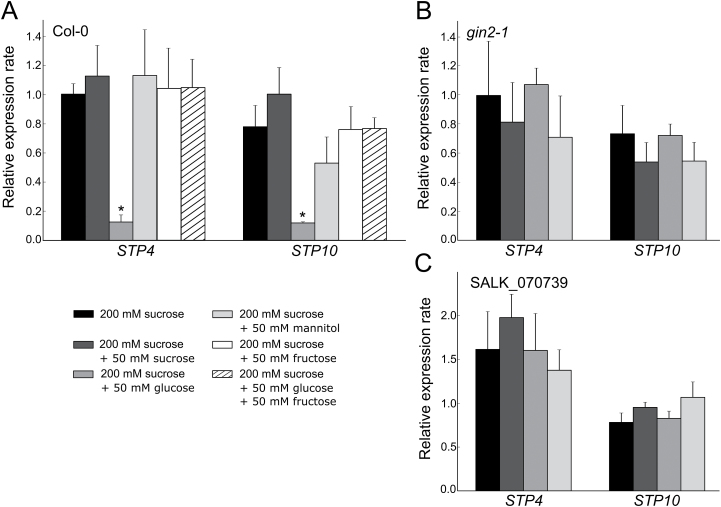
Analysis of *STP10* and *STP4* transcript levels in Col-0 and glucose insensitive pollen tubes germinated *in vitro* on media containing different carbohydrates. *STP10* and *STP4* transcripts were quantified by qPCR using total RNA extracted from Col-0, *gin2-1* or SALK_070739 pollen tubes grown *in vitro* for 5h on media containing either 200mM sucrose only or 200mM sucrose supplemented with 50mM of sucrose, glucose, mannitol, fructose or glucose+fructose. The diagram depicts expression ratios relative to *UBI10* under each growth condition. Means of three biological replicates±SE are shown. *P≤0.05 by Student’s *t*-test.

It has been reported that the expression of several *STP*s (*STP1*, *STP4*, *STP13* and *STP14*) is strongly repressed by glucose (Büttner *et al.*, 2000; Price *et al.*, 2004; Cordoba *et al.*, 2014). In order to analyse whether this transcriptional regulation applies also for *STP10*, pollen of Col-0 plants was germinated *in vitro* on media containing only 200mM sucrose or 200mM sucrose and glucose. Pollen tubes grown on a higher sucrose concentration or on medium supplemented with mannitol instead of glucose were used as controls. The relative expression rate of *STP10* was determined by qPCR (Fig. 6A). No significant difference between *STP10* expression in pollen tubes grown either on 200mM sucrose (black bars in Fig. 6), or 250mM sucrose (dark grey) or 200mM sucrose + 50mM mannitol (light grey) was observed. Though the expression was slightly reduced when the medium contained mannitol, this difference was not statistically significant. In contrast, addition of glucose to the medium (mid-grey) led to a significant decrease of *STP10* expression (only 12% compared with the medium containing 200mM sucrose). As the addition of another 50mM sucrose or of 50mM mannitol to the 200mM sucrose reference medium did not significantly alter *STP10* expression, the observed decrease of expression in the glucose sample is most likely not caused by osmotic effects but is a specific reaction to the presence of glucose. Fructose also did not lead to an *STP10* down-regulation in pollen tubes of WT plants, which emphasizes the specificity of the observed effect. However, the addition of both glucose and fructose abolished the glucose-mediated *STP10* repression (Fig. 6A). It has been demonstrated that the hexokinase1 (HXK1) complex acts as a glucose sensor and mediates glucose-dependent changes in gene expression in Arabidopsis (Cho *et al.*, 2006). To study a possible function of HXK1 in *STP10* repression, we isolated mRNA from pollen tubes of two independent *hxk* mutant lines grown on media with or without glucose. *gin2-1* (*glucose insensitive2-1*) contains a nonsense mutation and SALK_070739 carries a T-DNA insertion in the *HXK1* gene. In both lines, the mutation leads to the disruption of many glucose responses (Moore *et al.*, 2003; Aki *et al.*, 2007; Hsu *et al.*, 2014). qPCR analyses revealed no glucose-induced down-regulation of *STP10* in *gin2-1* (Fig. 6B) and in SALK_070739 pollen tubes (Fig. 6C). This indicates that the glucose-dependent regulation of *STP10* could be mediated via the HXK1 complex. It has been reported that another gene of the same gene family, *STP4*, is repressed by glucose (Price *et al.*, 2004; Cordoba *et al.*, 2014) and is also transcribed in pollen tubes (Wang *et al.*, 2008; Qin *et al.*, 2009). To study the role of HXK1 in the regulation of *STP4*, we furthermore determined the expression rates of *STP4*. As shown in Fig. 6A, the pattern obtained is very similar to *STP10* with almost no differences in expression between the sucrose, fructose and mannitol controls and a strong reduction of expression in the presence of glucose. Also for *STP4* the down-regulation by glucose disappeared in the *gin2-1* as well as in the SALK_070739 mutant, suggesting a shared molecular mechanism of transcriptional regulation.

### Characterization of a *stp10-1* T-DNA insertion line

**Fig. 7.**
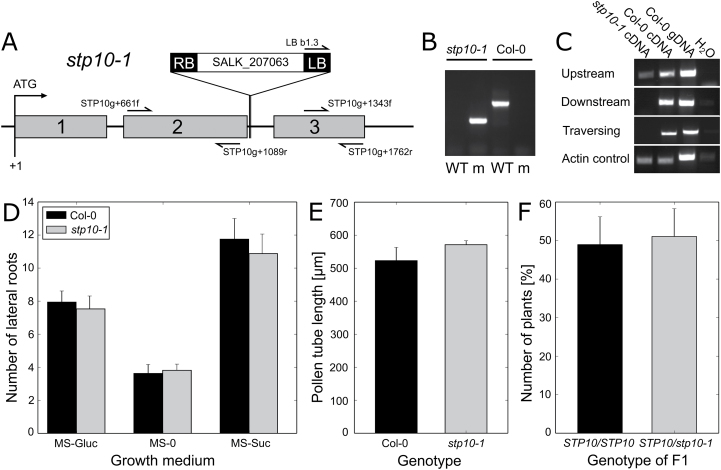
Characterization of the *stp10-1* T-DNA insertion line (SALK_207063). (A) Genomic organization of *STP10*. Exon regions containing coding sequences (grey bars) are numbered; introns and untranslated regions are shown as black lines. Arrows indicate the primers used in (B) and (C) and their orientation. The position of the T-DNA insertion with the orientation of the left border (LB) at the beginning of the second intron is marked. (B) PCR products obtained from genomic DNA preparations of a Col-0 and a homozygous *stp10-1* plant with the primer combinations STP10g+661fw/STP10g+1762r for the detection of the WT allele (WT) and LBb1.3/STP10g+1762r for the mutant allele (m). (C) PCR analyses of *STP10* cDNAs derived from pollen tube RNA of a homozygous *stp10-1* mutant plant and of a WT plant with primers amplifying the *STP10* sequence either traversing (STP10g+661f/STP10g+1762r) or upstream of (STP10g+661f/STP10g+1089r) or downstream of the insertion (STP10g+1343f/STP10g+1762r). WT genomic DNA was used as a control for genomic contaminations. PCR with *Actin2*-specific primers confirmed the presence of cDNA. (D– F) Phenotypic analyses of *stp10-1* mutant plants. (D) Number of side roots of 14-day-old *stp10-1* and WT seedlings on MS medium without sugars (MS-0), with 2% (w/v) glucose (MS-Gluc) or with 2% (w/v) sucrose (MS-Suc); *n*>20 for each sample. (E) Lengths of *stp10-1* and WT pollen tubes germinated *in vitro* for 6h. Mean values (±SE) of three biological replicates are shown (*n*>250 for each genotype in each experiment). (F) Genotypes regarding *STP10* in the F1 descendants of a cross-pollination experiment with WT pistils and pollen from a heterozygous *stp10-1/STP10* plant. Bars represent mean values (±SE) of the percentage of each genotype in the F1 generation of four independent crossings (*n*=100 in total). There were no statistical differences according to Student’s *t*-test.

To further analyse the physiological role of STP10 in pollen tubes and lateral root buds, an Arabidopsis line with a T-DNA insertion in the *STP10* gene (SALK_207063; Alonso *et al.*, 2003) was characterized. Sequencing of the mutant allele identified the exact position of the insertion to be 1175bp after the start codon at the beginning of the second intron (Fig. 7A). As no other mutant line for *STP10* has been described so far, we refer to this line as *stp10-1*. Plants homozygous for the *stp10-1* mutant allele were identified by PCR (Fig. 7B) and the complete loss of full-length *STP10* mRNA was confirmed by comparative RT-PCR analyses of pollen tube-derived total RNA from homozygous mutants and WT plants (Fig. 7C). As expected, truncated *STP10* mRNAs from the region upstream of the insertion could be detected also in the mutants. A possible translation of this partial mRNA would lead to truncated proteins lacking the predicted transmembrane helices IX–XII, which are therefore unlikely to form functionally active hexose transporters. Downstream of the insertion no mRNA fragment could be amplified by RT-PCR (Fig. 7C). Plants of the homozygous *stp10-1* line were analysed with respect to roots and pollen tubes, which are the identified expression sites of *STP10*. No differences compared with the WT could be detected concerning the number of lateral roots (Fig. 7D) and the length of the main root (Supplementary Fig. S2) on growth media containing sucrose, glucose or no sugar. The analysis of *stp10-1* pollen tubes also did not reveal any distinction in comparison with the WT: *in vitro* pollen growth assays revealed no significant difference in pollen germination rate (WT: 48%, *stp10-1*: 47%) or tube length (Fig. 7F). The *stp10-1* mutants were self-fertile and produced viable seeds; to compare the fertility of WT and mutant plants, a cross-pollination assay was performed. Pollen from a heterozygous *STP10*/*stp10-1* plant was used to pollinate WT pistils. The descendant generation showed a 50:50 segregation ratio of heterozygous and WT plants (Fig. 7E), indicating that WT and mutant allele are inherited equally. Hence, pollen tubes containing only the mutant allele in their haploid genome show the same fertility as WT pollen tubes expressing *STP10*. This confirms that the mutation of *STP10* seems not to interfere significantly with pollen viability, tube growth or fertility.

## Discussion

The present paper describes the functional analysis and *in planta* localization of *STP10*, a previously uncharacterized member of the Arabidopsis MST(-like) superfamily.

Expression of *STP10* in the heterologous yeast system characterized the encoded protein as a high-affinity, energy-dependent H^+^–monosaccharide symporter that accepts glucose, galactose, and mannose as substrates. This group of substrates is shared by most other STPs with the exception of STP9, which is specific for glucose (Schneidereit *et al.*, 2003). In contrast to many other STPs like for example STP1–4 and STP11 (Büttner, 2010), STP10 did not mediate the uptake of xylose. The *K*_M_ value for glucose lies within the micromolar range (7.6±1.7 µM) and thus is in line with the values measured for all other STPs characterized so far (Sauer *et al.*, 1990; Truernit *et al.*, 1996; Truernit *et al.*, 1999; Büttner *et al.*, 2000; Scholz-Starke *et al.*, 2003) with the exception of STP3, which has a lower affinity to glucose (Büttner *et al.*, 2000).

Expression of *STP10-GFP* in Arabidopsis protoplasts showed a clear localization of the fusion protein at the plasma membrane, which is the reported subcellular localization of all characterized STPs (Büttner, 2010). The tonoplast obviously is no target membrane for STPs.

Promoter–reporter gene analyses demonstrated *STP10* expression predominantly in pollen tubes, and, weaker, also in emerging side roots and in the adventitious root meristem. Both the male gametophyte and roots are sink cells or sink organs, which rely on carbon supply. Hence, *STP10* expression is sink specific, which is again a common feature of the *STP* gene family (Büttner, 2010) with the exception of *STP3* (Büttner *et al.*, 2000) and *STP14* (Poschet *et al.*, 2010), which are additionally expressed in leaves. A comparison of *pSTP10:GUS* and *pSTP10:STP10g-GUS* plants indicated that the restriction of *STP10* expression to sink tissues is mediated by intragenic regions, most likely the introns. Whereas staining of *pSTP10:STP10g-GUS* plants only marked pollen tubes, emerging side roots and the adventitious root meristem, the reporter plants lacking the genomic sequence showed also a strong staining in other tissues, but only a weak staining of pollen tubes. In general, a *promotor:GUS* construct reflects gene expression driven by the promoter while a construct that includes the genomic sequence of the gene of interest provides a read-out of the presence of the protein as well. Our data strongly suggest that the *STP10* gene may be expressed in more cells than those in which the protein is actually made. The genomic sequence of *STP10* obviously has two regulatory effects. It is both inhibiting the expression of the gene in all tissues but pollen tubes and roots and enhancing its expression in pollen tubes. A non-linear correlation between promoter activity and protein synthesis and the contribution of introns to a strong expression have been reported for many genes of Arabidopsis and other plants (Fiume *et al.*, 2004; Rose, 2004). For most genes the presence of the introns leads to an expression in more tissues (Jeong *et al.*, 2007). In contrast, an intron-mediated decrease of expression in certain tissues as observed for *STP10* has only been described for the *AGAMOUS* gene (Sieburth and Meyerowitz, 1997).

Besides *STP10*, *STP1*, *STP4*, *STP7*, and *STP13* are also predicted to be significantly expressed in root tissues (Büttner, 2010), but only for *STP1* and *STP4* has this expression been confirmed by further analyses (Truernit *et al.*, 1996; Sherson *et al.*, 2000). STP1 can mediate the uptake of external hexoses (Sherson *et al.*, 2000) and microarray data indicate expression in the whole root (Büttner, 2010). *STP4* is only expressed in root tips and older side roots (Truernit *et al.*, 1996), and not in emerging buds of lateral roots or the adventitious root meristem. The expression of *STP10* in tissues not covered by *STP4* expression indicates that STP10 might take over the function of STP4 in these regions. But most likely the STPs expressed in roots have at least partially redundant functions. As non-photosynthetic but fast growing tissues, the root tips, lateral roots and adventitious root meristem all rely on sugar supply from the phloem, especially as roots are reported to store almost no starch (Tsai *et al.*, 2009). It was shown that the unloading of sucrose from the phloem in roots occurs mainly via the symplast (Oparka *et al.*, 1994). However, the high expression of the cell wall invertase gene *cwINV1* (Büttner, 2010) in parallel with at least three *STP*s indicates that the distribution of photoassimilates in the root could be mediated by a combination of symplastic and apoplastic transport. The transport of sugars via both the apoplastic and the symplastic routes could increase the total amount of sugar delivered to rapidly growing root parts. This hypothesis is supported by the fact that symplastic diffusion of sugars from the phloem cannot cover all the carbon requirements of the root meristem in maize (Bret-Harte and Silk, 1994). Furthermore, cell wall invertases and STPs could also have some kind of retrieval function: sucrose lost from the cells could be cleaved and re-imported via monosaccharide transporters.

The *STP10* promoter activity in roots was visible only in transgenic plants that synthesized the reporter protein GUS, which also allows detection of weak promoter activity. In pollen tubes, however, both GUS and GFP were detectable. This indicates a stronger expression of *STP10* in pollen tubes compared with roots.

In contrast to roots, pollen tubes are symplastically isolated. Although the pollen grains are preloaded with nutrients during their development, the rapid elongation of growing pollen tubes is an energy-dependent process and probably requires the uptake of additional carbohydrates from the surrounding tissue. The callose in the cell wall of pollen tubes is synthesized from UDP-glucose (Chen and Kim, 2009), which requires additional energy. The necessity of sugar uptake into germinating pollen and growing pollen tubes is underlined by the presence of several sugar transporters in pollen tubes.

In addition to *STP10*, four other monosaccharide transporter genes, *STP4*, *STP6*, *STP9*, and *STP11*, are expressed in pollen tubes, which indicates a high functional redundancy (Schneidereit *et al.*, 2005; Büttner, 2010). This redundancy also explains the absence of an obvious phenotype in the *stp10-1* knockout plants. *STP11* was proposed to be one of the highest expressed genes in pollen tubes (Schneidereit *et al.*, 2005; Wang *et al.*, 2008; Qin *et al.*, 2009; Büttner, 2010). Strong expression levels were also predicted for *STP10* and *STP4* in pollen tube transcriptome data (Wang *et al.*, 2008; Qin *et al.*, 2009). All encoded STP proteins were found in germinating pollen tubes, even if mRNAs of *STP4*, *STP6*, *STP9*, and *STP11* accumulate already in mature pollen prior to pollen germination (Truernit *et al.*, 1996; Scholz-Starke *et al.*, 2003; Schneidereit *et al.*, 2003; Schneidereit *et al.*, 2005; Büttner, 2010). In contrast, our RT-PCR and reporter gene data suggest that *STP10* mRNA is very weakly expressed or even absent in dry pollen and strongly induced during pollen tube growth. This is consistent with pollen tube transcriptome data, which showed a strong increase of *STP10* mRNA in pollen tubes after germination (Qin *et al.*, 2009; Leydon *et al.*, 2013). In addition, *in vitro* grown pollen tubes showed strong GUS and GFP labelling indicating that *STP10* gene induction did not depend on signals from the stigma, which again coincides with transcriptome data from *in vitro* or semi-*in vivo* grown pollen tubes (Qin *et al.*, 2009; Leydon *et al.*, 2013). Interestingly, mutants in three MYB transcription factors fail to induce *STP10* expression in pollen tubes, which indicates a function of these factors in *STP10* induction during pollen germination (Leydon *et al.*, 2013). The analysis of *STP4* and *STP10* transcript levels has shown that both genes are down-regulated by glucose. A glucose-dependent down-regulation has been suggested for several *STP*s. It was recently analysed in more detail for *STP1* (Cordoba *et al.*, 2014). The glucose-dependent down-regulation of *STP4* and *STP10* is missing in two independent *hexokinase1* mutant lines, indicating that these two genes could be regulated on the transcriptional level via the glucose sensor HXK1. However, for *STP1* it was reported that its sugar-dependent regulation is independent of HXK1 (Cordoba *et al.*, 2014), suggesting that there are different pathways for sugar-dependent regulation of *STP*s. Additionally we showed that the supply of fructose instead of glucose in the pollen tube growth medium did not induce *STP10* down-regulation. This is consistent with recent data, which showed that HXK1 is not involved in fructose signalling in Arabidopsis, although this enzyme carries out metabolic activities for both glucose and fructose. The authors suggested that this is due to the fact that HXK1 has an approximately 100-fold higher affinity for glucose compared with fructose (Gonzali *et al.*, 2002; Cho and Yoo, 2011). Fructose signalling responses in Arabidopsis are instead mediated by FRUCTOSE INSENSITIVE1 (FINS1/FBP), a putative FRUCTOSE-1,6-BISPHOSPHATASE (Cho and Yoo, 2011). Interestingly, we observed that the glucose-mediated down-regulation of *STP10* was abolished when both sugars, fructose and glucose, were present in the medium. The latter observation could explain why no glucose-mediated *STP10* down-regulation was detectable in medium containing only sucrose, which could be cleaved by cell wall invertases into glucose and fructose (Hirsche *et al.*, 2009). A crosstalk between fructose signalling mediated by FINS1/FBP and glucose signalling mediated by HXK1 has been postulated for early seedling establishment in Arabidopsis (Cho and Yoo, 2011). Possible interactions between both sugar signalling pathways during pollen tube growth will require additional analyses of *fins1/fbp* pollen tubes in future research.

So far it cannot be concluded that the observed regulation of *STP10* is directly mediated by the HXK1 complex, since it could also occur through a more indirect pathway. However, the observation that the glucose sensor HXK1 could have a regulatory function in the highly energy-consuming growth of pollen tubes is an interesting aspect for the general question of pollen tube energy supply. Therefore, detailed studies of HXK1 in pollen tubes should be the focus of future research interests.

In addition to monosaccharide transporter genes at least five sucrose transporter genes, *SUC1*, *SUC3*, *SUC7*, *SUC8*, and *SUC9*, are expressed in pollen grains or tubes (Stadler *et al.*, 1999; Meyer *et al.*, 2004; Qin *et al.*, 2009; Leydon *et al.*, 2013; Leydon *et al.*, 2014). The sucrose transporter SUC1 was reported to be necessary for efficient pollen germination both *in vivo* and *in vitro*. *suc1* knockout pollen displayed a reduced fertility in segregation analyses, even if the number of seeds in homozygous *suc1* knockout plants was reported not to be reduced compared with wild type (Sivitz *et al.*, 2008).

Apart from *STP*s and *SUCs*, Arabidopsis pollen additionally expresses the cell wall invertase gene *cwINV2* (Hirsche *et al.*, 2009). It has not been studied in detail whether cwINV2 is active only in developing pollen or also in growing pollen tubes. However, pollen tubes show better *in vitro* germination on medium containing sucrose. Hence, if sucrose is provided by the transmitting tissue, the disaccharide could be cleaved by invertases, transported into the pollen tube via plasma membrane located STPs and used as an energy source. The overlapping expression of at least five *STP*s and five *SUC*s in pollen tubes indicates that they have at least partially redundant functions to ensure that the male gametophyte is in any case provided with enough nutrients to complete its journey and fertilize the egg cell. The cleavage of sucrose by invertases and the following uptake of monosaccharides could contribute to a quick reduction of the sucrose concentration in the transmitting tract. This would increase the concentration gradient between source and sink and thereby promote the long-distance transport of sucrose from the source leaves to the transmitting tract.

The parallel expression of low-affinity but highly efficient *SUC*s and high-affinity but low-capacity *STP*s, some of which are induced under low glucose conditions, could be explained by the following hypothesis: under normal conditions, the pollen tube mainly subsists on sucrose; a low glucose concentration in the apoplast of the transmitting tract could be a starvation signal and lead to the induction of high-affinity STPs to gather as much sugar as possible. Alternatively, glucose could be a general signal molecule for pollen tube growth and guidance rather than a nutrient. Decreasing glucose concentrations require a higher expression of the glucose uptake system to ensure the uptake of this signal.

The presented data shed light on the important question of how pollen tubes regulate sugar uptake in response to external signals and how they make sure that their carbon demand is covered. However, it has not been analysed so far whether monosaccharides and sucrose are indeed present in the apoplast of the ovary, and if the uptake of either sucrose or glucose or both is essential for pollen tube growth *in vivo*. The analysis of double/multiple *stp* and *suc* knockout plants will be necessary to elucidate the physiological role of both transporter types and their respective sugar substrates for pollen development, germination and tube growth.

## Supplementary data

Supplementary data are available at *JXB* online.

Figure S1. Confocal images of the subcellular localization of STP10-GFP in *Arabidopsis* protoplasts

Figure S2. Length of main roots of the *stp10-1* T-DNA insertion line

Supplementary Data
