# Supplementary material for: STP10 encodes a high-affinity monosaccharide transporter and is induced under low-glucose conditions in pollen tubes of Arabidopsis
Source: J Exp Bot. 2016 Feb 18;67(8):2387–99. doi: 10.1093/jxb/erw048 (PMC4809294; doi:10.1093/jxb/erw048)
Supplement: Supplementary Data [file supp_67_8_2387__index.html]

STP10 encodes a high-affinity monosaccharide transporter and is induced under low-glucose conditions in pollen tubes of Arabidopsis — STP10 encodes a high-affinity monosaccharide transporter and is induced under low-glucose conditions in pollen tubes of Arabidopsis — Supplementary Data 

# *STP10* encodes a high-affinity monosaccharide transporter and is induced under low-glucose conditions in pollen tubes of Arabidopsis

## Supplementary Data

Data files

- supplementary\_figures\_S1\_S2.pdf - Supplementary Data
