# Supplementary material for: STP10 encodes a high-affinity monosaccharide transporter and is induced under low-glucose conditions in pollen tubes of Arabidopsis
Source: J Exp Bot. 2016 Feb 18;67(8):2387–99. doi: 10.1093/jxb/erw048 (PMC4809294; doi:10.1093/jxb/erw048)
Supplement: Supplementary Data [file supp_erw048_supplementary_figures_S1_S2.pdf]

STP10 encodes a high-affinity monosaccharide transporter and is induced under low-glucose conditions in pollen tubes of *Arabidopsis*

Theresa Rottmann, Wolfgang Zierer, Christa Subert, Norbert Sauer and Ruth Stadler

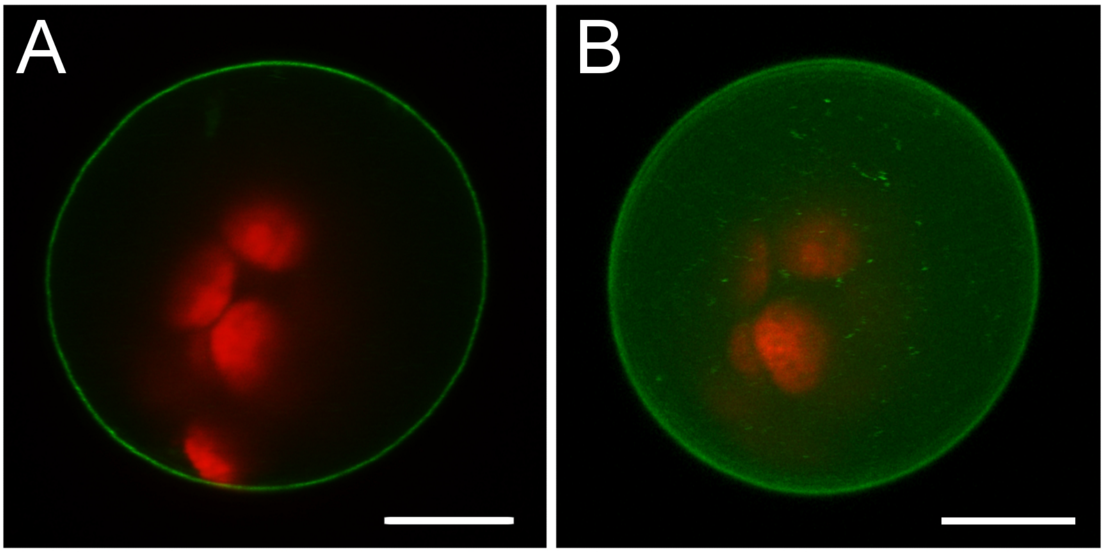

Supplementary figure 1: **Confocal images of the subcellular localization of STP10-GFP in *Arabidopsis* protoplasts.** (A) Single optical section of a mesophyll protoplast expressing *STP10c-GFP* under the control of the 35S promoter. (B) Maximum projection of a protoplast transformed with the same construct. GFP is shown in green, chlorophyll autofluorescence in red. Scale bars: 10 μm.

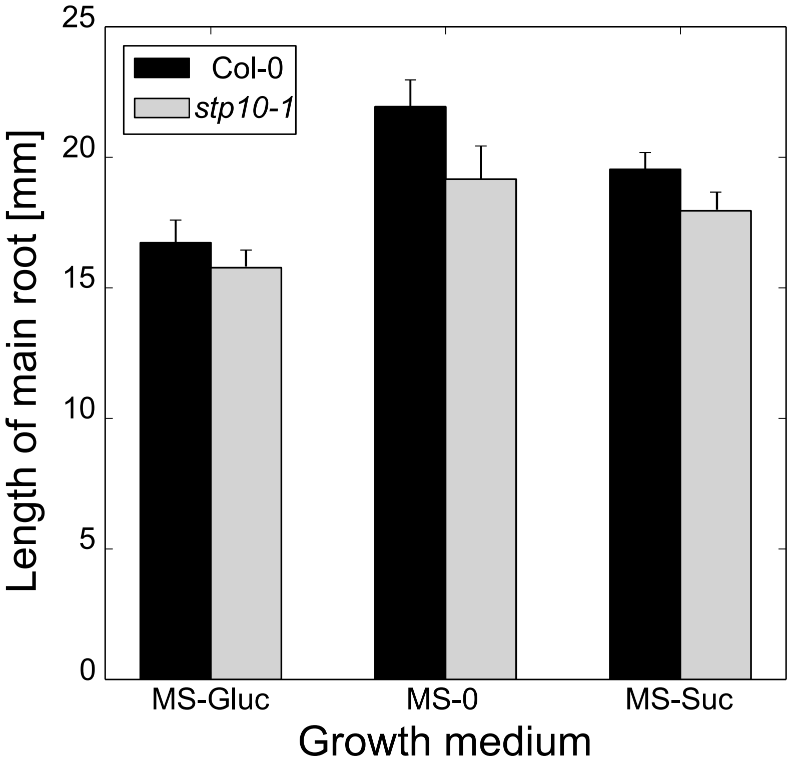

Supplementary figure 2: **Length of main roots of the *stp10-1* T-DNA insertion line (SALK_207063).** Mean ± SE of the main root length of 14-day-old *stp10-1* and WT seedlings on MS medium without sugars (MS-0), with 2% (w/v) glucose (MS-Gluc) or with 2% (w/v) sucrose (MS-Suc); $n > 20$ for each sample. No statistical differences according to Student's t-test.
